# Spreading predictability in complex networks

**DOI:** 10.1038/s41598-021-93611-z

**Published:** 2021-07-12

**Authors:** Na Zhao, Jian Wang, Yong Yu, Jun-Yan Zhao, Duan-Bing Chen

**Affiliations:** 1 Electric Power Research Institute of Yunnan Power Grid Co., Ltd, Kunming, 650200 People’s Republic of China; 2 Key Laboratory in Software Engineering of Yunnan Province, School of Software, Yunnan University, Kunming, 650504 People’s Republic of China; 3 School of Computer Science and Engineering, University of Electronic Science and Technology of China, Chengdu, 611731 People’s Republic of China; 4 College of Information Engineering and Automation, Kunming University of Science and Technology, Kunming, 650217 People’s Republic of China; 5 Beijing Special Vehicle Institute, Beijing, 100072 People’s Republic of China; 6 The Research Base of Digital Culture and Media, Sichuan Provincial Key Research Base of Social Science, Chengdu, 611731 People’s Republic of China; 7 Union Big Data, Chengdu, 610041 People’s Republic of China

**Keywords:** Information theory and computation, Statistical physics, thermodynamics and nonlinear dynamics

## Abstract

Many state-of-the-art researches focus on predicting infection scale or threshold in infectious diseases or rumor and give the vaccination strategies correspondingly. In these works, most of them assume that the infection probability and initially infected individuals are known at the very beginning. Generally, infectious diseases or rumor has been spreading for some time when it is noticed. How to predict which individuals will be infected in the future only by knowing the current snapshot becomes a key issue in infectious diseases or rumor control. In this report, a prediction model based on snapshot is presented to predict the potentially infected individuals in the future, not just the macro scale of infection. Experimental results on synthetic and real networks demonstrate that the infected individuals predicted by the model have good consistency with the actual infected ones based on simulations.

## Introduction

Spreading dynamics is an important issue in spread and control^1,2^ of rumor^3,4^ and disease^5–8^, marketing^9^, recommending^10–12^, source detecting^13,14^, and many other interesting topics^15–18^. Generally speaking, we can not observe the transmission process of infectious diseases, but can only observe the snapshot at a certain time. How to predict the infection probability^19^, infection scale^20,21^, or even the infected nodes precisely from a given snapshot has been gotten much attention in recent years.

Researchers have gotten many achievements on macro level of spread such as phase transition of spread^22^ and basic reproduction number^23^. Up to now, many researches focus on estimating of infection scale. The simplest one is mean-field model, in which, the spread coverage can be predicted by using differential equations^20^. Besides mean-field model, some more realistic models such as pair approximation^21^ and permutation entropy^24^ are considered to predict the infection scale or infectious disease outbreaks. The main difference between mean-field and pair approximation is that the former(latter) approximates high-order moments in term of first (second) order ones. Researchers studied the predictability of a diverse collection of outbreaks and identified a fundamental entropy barrier for disease time series forecasting through adopting permutation entropy as a model independent measure of predictability^24^. Funk et al.^25^ presented a stochastic semi-mechanistic model of infectious disease dynamics that was used in real time during the 2013–2016 West African Ebola epidemic to fit the simulated trajectories in the Ebola Forecasting Challenge, and to produce forecasts that were compared to following data points. Zhang et al.^26^ proposed a measurement to state the efforts of users on Twitter to get their information propagation. They found that small fraction of users with special performance on participation can gain great influence, while most other users play an intermediate role during the information propagation.

Up to now, most researches focus on macro level of spreading prediction. Besides analysis on macro level, we also should pay attention to the details of infected individuals so as to contain the spread of serious infectious diseases such as SARS^27^, H7N7^28^ and COVID-19^29^. Chen et al. did some interesting works on this area^19^. They presented an iterative algorithm to estimate the infection probability of the spreading process and then to predict the spreading coverage from a given snapshot. In this report, we present a probability based prediction model to estimate the probability of a node to be infected, further, to determine the potentially infected nodes in the future rather than macro scale.

**Figure 1:**
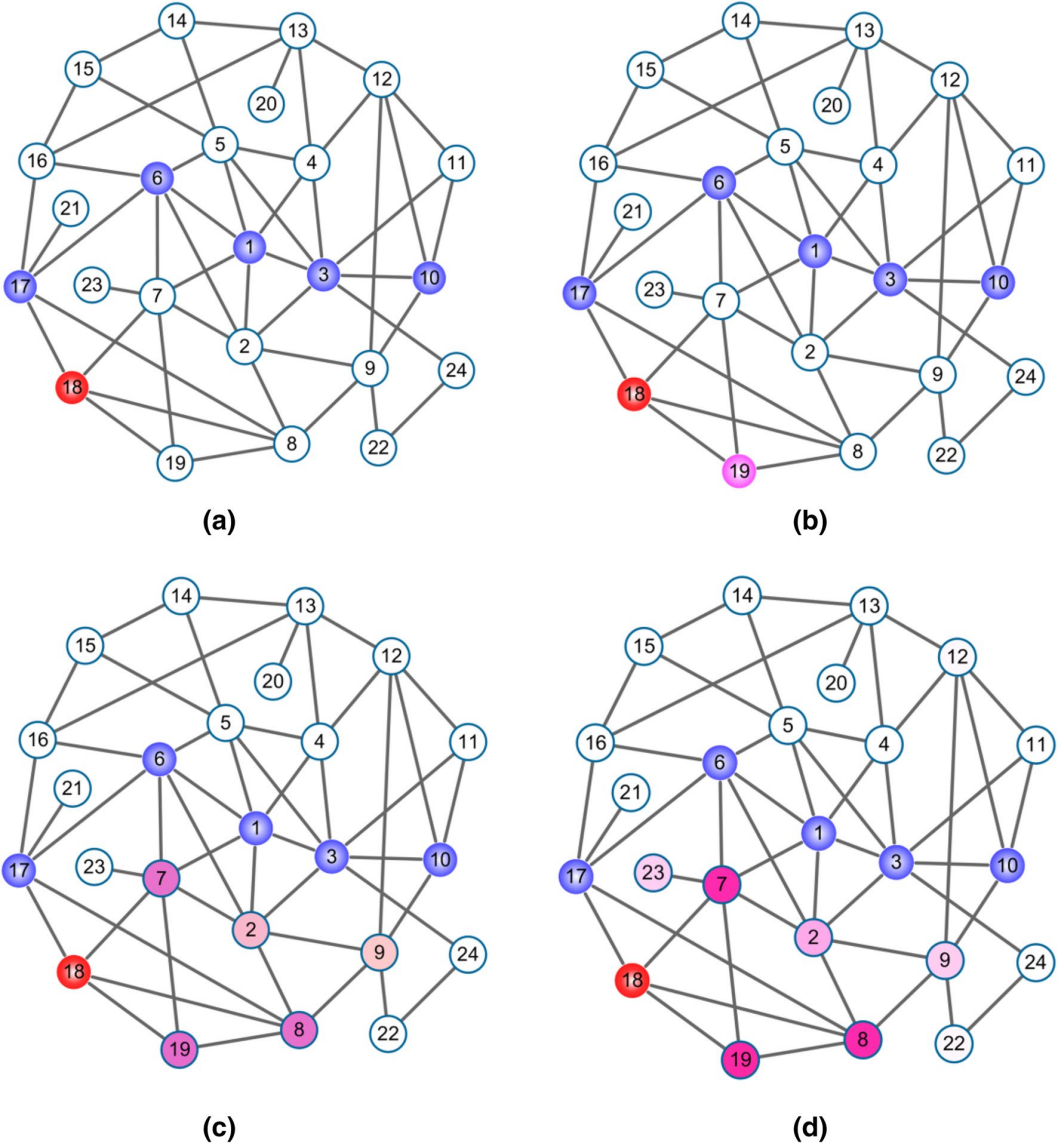
A toy network with 24 nodes. (**a**) The snapshot includes 5 recovered nodes, i.e., 1, 3, 6, 10, 17, and 1 infected node, i.e., node 18, (**b**) a certain spreading simulation result from snapshot, only node 19 is infected when spreading achieves steady state, (**c**) average result on 10000 simulations from snapshot, and (**d**) result of probability prediction model from snapshot. In (**c**,**d**), the shades of nodes indicate the probability to be infected when spreading achieves steady state.

Figure 1 is a toy network with 24 nodes. The snapshot includes 5 recovered nodes and 1 infected node, as shown in Fig. 1a. A certain spreading simulation result, average result on 10000 simulations, and result of probability prediction model from snapshot are shown in Fig. 1b–d respectively. From this toy network, it can be seen that the result obtained by the probability prediction model is consistence with that by the average over 10000 simulations very well, that is, nodes 7, 8, and 19 have high probability to be infected, nodes 2 and 9 have middle probability to be infected, while other nodes have relatively low probability to be infected, as shown in Fig. 1c,d.

## Results and discussion

We evaluate the model on synthetic and real networks. Synthetic networks are Wattes–Strogatz (WS) networks^30^, Barabási–Albert (BA) networks^31^ and Given-Newman (GN) community networks^32^. Each synthetic network has 4000 nodes and each GN community network has 40 communities. Eleven real networks are cond-mat, astro-Ph, email, c.elegens, ecoli, internet, PGP, TAP, HEP, Y2H and power. The number of nodes and edges are listed in Table 1.

**Table 1:** The properties and analyzing results on 11 real networks.

Networks	#Nodes	#Edges	$\rho$	$N_I$
cond-mat	39577	175693	0.9430	0.0152
astro-Ph	16046	121251	0.9426	0.0575
email	1133	5451	0.9860	0.0628
c.elegens	453	2025	0.9900	0.1143
ecoli	230	695	0.9558	0.0509
internet	22963	48436	0.9541	0.0625
PGP	10680	24316	0.8074	0.0069
TAP	1373	6833	0.5897	0.0101
HEP	7610	15751	0.5975	0.0016
Y2H	1846	2203	0.3214	0.0016
power	4941	6594	0.2762	0.0003

In order to evaluate the model, we employ the Susceptible-Infected-Removed (SIR) model^33^ to simulate the spreading process on networks. In a network, we randomly select one node as the initial spreader. The information from this node will infect each of its susceptible neighbors with probability $\mu$. For simplicity, we assume that the node will immediately recover (i.e., the recovering probability is 1) after infecting neighbors. Of course, if the recovering probability is less than 1, it can be analyzed similarly, we will study this in the future. The new infected nodes continue to infect their neighbors in next step. If it is not specially stated, we take the snapshot after five steps of spreading from the initial node as the known information.

The correlations $\rho$ on 11 real networks are shown in Table 1, where $\rho$ is the Pearson correlation between the results of prediction and actual ones based on simulations. From Table 1, it can be seen that the results obtained by prediction model are good consistency with actual results based on simulations, especial for the case of large number of infected nodes $N_I$ of snapshot. It is noted that the correlation $\rho$ and $N_I$ have strong positive correlation. For networks Y2H and power, the correlation $\rho$ is extremely low since $N_I$ is very small. Actually, in these cases, there are few infected nodes in snapshot. Furthermore, the networks are very sparse, so, it is hard to predict the nodes being infected from snapshot in the future. While for networks cond-mat, astro-Ph, email, c.elegens, ecoli and internet, the correlations $\rho$ are larger than 0.9, this indicates that the infected individuals predicted by model are basically consistent with the actual ones based on simulations.

Moreover, we also deeply analyze the effect of some parameters on the prediction model by using synthetic networks, including: (1) the effect of infection probability, (2) the effect of network structure, and (3) the effect of stage of snapshot.

### The effect of infection probability

**Figure 2:**
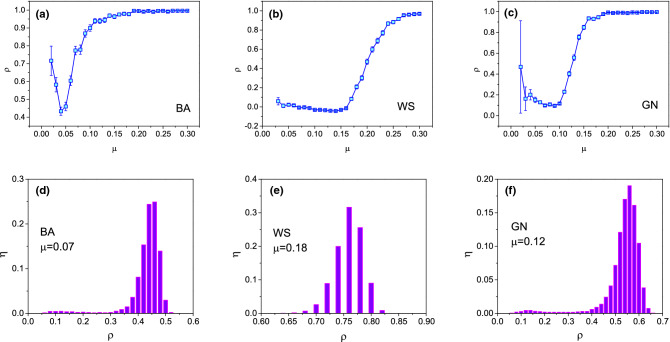
The correlation $\rho$ under different infected rate $\mu$ on (**a**) BA, (**b**) WS and (**c**) GN networks. The distribution $\eta$ of correlation $\rho$ are shown in (**d**) BA, (**e**) WS and (**f**) GN, where $\eta$ is the ratio of the number of snapshots whose correlation $\rho$ located in a certain interval with width being 0.02 to the total of snapshots. The network parameters are $N=4000, \langle k\rangle =10,p=0.1$ for WS network, $N=4000, \langle k\rangle =10$ for BA network, and $N=4000,\langle k\rangle =10,\langle k_{in}\rangle =7$ for GN network. The error bar in (**a**–**c**) and the distribution of correlation $\rho$ in (**d**–**f**) are obtained by the results under 200 snapshots.

Figure 2 shows the Pearson correlation $\rho$ between the results of averaging on 200 simulations and that of probability prediction model under different infection probability $\mu$ on WS, BA and GN networks. Generally, the correlation get larger while $\mu$ getting larger. For large $\mu$, e.g., $\mu =0.3$, the correlation approach to 1 since most of nodes will be infected. From Fig. 2, it can be seen that there exists a transition point, in detail, the transition point at $\mu =0.15$ for WS network (see Fig. 2b) and at $\mu =0.1$ for GN network (see Fig. 2c). This can be explained as follows: the information almost do not diffuse if $\mu$ is small ($\mu <0.15$ for WS networks and $\mu <0.1$ for GN network), and the infected nodes are highly random for different simulations. It is noted that there hardly exist transition point in BA network. It can be explained as follows: the information will easily reach to the node with large degree regardless the location of initially infected node, eventually, reach to other nodes for its heterogenous structure. Interestingly, if $\mu$ is very small (e.g., $\mu =0.02$), the correlation is getting large in BA network, as shown in Fig. 2a. Actually, for very small $\mu$, only a few snapshots in 200 simulations can be utilized to analyze the correlation $\rho$ since spread stops in two or three steps in most simulations, the results have no statistical significance. Besides, the distribution of correlation $\rho$ under the results of 200 independent runs are listed in Fig. 2d–f. From these three subfigures, it can be seen that the distributions of correlation $\rho$ of BA and GN networks are similar, while that of WS network are generally large comparing with BA and GN networks.

### The effect of network structure

**Figure 3:**
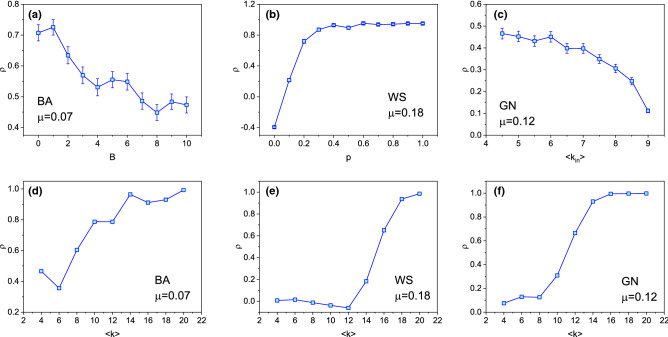
The correlation $\rho$ for three types of networks with different structural parameters. In (**a**), B is a tunable parameter while generating network, (**b**) *p* is the rewiring probability, (**c**) $\langle k_{in}\rangle$ is the average internal degree, and (**d**–**f**) $\langle k\rangle$ is the average degree.

Figure 3 shows the correlation for three types of networks with different structural parameters. For WS network, we study the effect of the rewiring parameter *p* on correlation. For BA network, we consider a variant form of it in which each new node *u* connects to an existing node *v* with probability $p_u=(k_u+B)/\sum _v(k_v+B)$^34,35^. This modified model allows a selection of the exponent of the power-law scaling in the degree distribution $p(k)\sim k^{-\gamma }$ with $\gamma =3+B/m$ in the thermodynamic limit where *m* is the number of nodes should be connected when a new node is added and *B* is tunable parameter. With this network, we study the effect of *B* on correlation. For GN network, we study the effect of $\langle k_{in}\rangle$ on correlation, where $\langle k_{in}\rangle$ is the average internal degree of nodes in community. For a node *u* in community *C*, its internal degree $k_{u}^{in}$ can be written as:

1 $$\begin{aligned} k_u^{in}=\sum _{u,v}\delta _{u,v}, \end{aligned}$$

$\delta _{u,v}=1$ if *v* is also in community *C*, otherwise $\delta _{u,v}=0$. For standard BA network, i.e., $B=0$, there are a few nodes with extremely large degree, the information can be spread out easily so long as it reaches to a node with large degree. So, it is relatively easy to predict which node will be infected in the future. As *B* increasing, the network evolves to random, a node getting infected or not will be hard to predict relatively, so the correlation decreases when *B* increases, as shown in Fig. 3a. In WS network, if rewiring probability $p<0.2$, the information hardly diffuse to other nodes since the WS network is almost regular, so it is hard to predict the infected nodes. As rewiring probability *p* getting larger, the network getting more random, the information reaches to other nodes easily, consequently, it is easy to predict the infected nodes, as shown in Fig. 3b. In GN network, if average internal degree $\langle k_{in}\rangle$ is larger, the community structure is clearer, correspondingly, the information is harder to escape the community boundary, and the correlation will getting worse, as shown in Fig. 3c.

Besides the network parameter listed above, the density of network, i.e., average node degree $\langle k\rangle$, also affects the correlation, as shown in Fig. 3d–f. It can be seen that the correlation is small for small average node degree $\langle k\rangle$. Especially in WS and GN networks, for a large scope of average node degree ($\langle k\rangle <12$ in WS and $\langle k\rangle <8$ in GN), the correlation is extremely small, there exists an obvious transition points, as shown in Fig. 3d,f. Actually, in WS and GN networks, when the average degree is small, the snapshot contains few infected nodes, so, little usable information can be used to predict. This leads to the inaccurate estimation of $\mu$^19^, further, the prediction of subsequent infected nodes is also inaccurate. In fact, it can be seen from Table 1 that if the number of infected nodes $N_I$ is small, the correlation $\rho$ is also low. We will study the essential reason of this issue in the future.

### The effect of stage of snapshot

**Figure 4:**
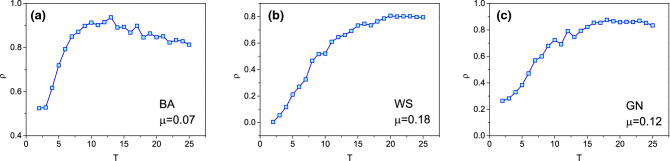
The correlation $\rho$ under different stage of snapshot. Smaller *T* indicates earlier stage and larger *T* indicates latter stage.

We further analyze the correlation $\rho$ under different stage of snapshot, as shown in Fig. 4. In Fig. 4, *T* is the spreading steps of snapshot. Generally, it is difficult to estimate the infected rate precisely if just the snapshot in the early stage is given since there is little usable information, so, it is hard to predict the infected nodes. As *T* increases, more information could be used, the correlation $\rho$ is getting larger. In the late stage, many nodes of snapshot are infected or recovered, the left nodes are hard to be infected, so the correlation $\rho$ are getting smaller, especially in BA network since most of all nodes are recovered.

From Figs. 3 and 4, it is interesting that the prediction results fluctuate greatly for different parameters, while the fluctuation of the results is very small under determined parameter. For example, as shown in Fig. 3b, if the reconnection parameter *p* is small, the correlation $\rho$ is low. However, if the reconnection parameter *p* is high, the correlation $\rho$ is high. No matter what the value of *p*, the error bar is small under a certain *p*, this indicates that the correlation on determined parameter *p* has little change. In Fig. 4, although the correlation fluctuates greatly with stage of the snapshot, it changes very little for a determined snapshot.

In conclusion, this report mainly predicts the potential infected individuals according to the currently observed snapshot, which has significance on prevention and control of infectious diseases such as COVID-19. Due to the popularity of mobile device, it is relatively easy to obtain users’ contact network, which provides certain basic conditions of prediction on potential infected individuals.

## Methods

For a given snapshot, we use IAIP^19^ method to estimate the infection probability. In IAIP model, we denote the number of infected nodes as $N_I$, the number of susceptible nodes as $N_S$ and the number of recovered nodes as $N_R$. $N_S+N_I+N_R=N$ since we use SIR spreading model. If a susceptible node *j* contacts an infected node *i*, node *j* has an opportunity to be infected. For an infected node *i* at step *t* (recovered at step $t + 1$), the contact times with its susceptible neighbors are $k_i-m_i$ ($m_i$ neighbors have been infected before step *t*). So, the total contact times before step *T* are $\sum _{i\in R}k_i-m_i$ where *T* is the spreading steps of snapshot. In these $\sum _{i\in R}k_i-m_i$ contacts, $N_R + N_I-1$ nodes are infected, so the infection probability $\mu$ can be approximately calculated by:

2 $$\begin{aligned} \mu =\frac{N_R+N_I-1}{\sum _{i\in R}(k_i-m_i)}, \end{aligned}$$

where $k_i$ is the degree of node *i* and $m_i$ is the number of infected or recovered nodes in the neighbors of node *i* when it is infected at step *t* ($t<T$ where *T* is the spreading steps of snapshot). Since the exact value of $m_i$ cannot be directly extracted from the snapshot, we use its expected value ${\overline{m}}_i$ to approximate. Actually, ${\overline{m}}_i$ is the weighted average of $M_i$ states where each state $S_l(1 \leqslant l \leqslant M_i)$ has exactly *l* neighbors having been infected before node *i* is infected. When node *i* is infected, the probability that exactly one neighbor has been infected is $\mu$ and the probability that exactly two neighbors have been infected is $\mu \cdot (1-\mu )$. Generally, the probability that $l(1 \leqslant l \leqslant M_i)$ neighbors have been infected is $\mu \cdot (1- \mu )^{ l-1}$ where $M_i$ is the total number of infected or recovered nodes of $i^{\prime }$s neighbors in the snapshot. Moreover, the number of infected or recovered neighbors will not exceed $M_i$ when *i* is infected. So, the probability that exactly $l(1\le l\le M_i)$ neighbors have been infected is approximated by the normalized value of $\mu \cdot (1-\mu )^{q-1}(1\le q \le M_i)$. Based on these, the expected value ${\overline{m}}_i$ of recovered or infected neighbors when *i* is infected can be calculated by the weighted average of $l(1\le l\le M_i)$ , that is:

3 $$\begin{aligned} m_i\approx {\overline{m}}_i=\frac{\sum _{l=1}^{M_i}l\cdot \mu \cdot (1-\mu )^{l-1}}{\sum _{q=1}^{M_i}\mu \cdot (1-\mu )^{q-1}}=\frac{\sum _{l=1}^{M_i}l\cdot \mu \cdot (1-\mu )^{l-1}}{1-(1-\mu )^{M_i}}, \end{aligned}$$

On the basis of Eq. 2 and Eq. 3, $\mu$ and $m_i$ are expected to respectively approach their true values. In real situation, it is very difficult to estimate $m_i$ accurately since we just can obtain the information at time *T* from snapshot. In the future study, we will combine other strategies such as source detection from snapshot^13,36^ to estimate $m_i$ more accurately.

In the proposed model, a group of infected individuals try to infect a node *i* until it is infected. Actually, we hold a reactive process in this report since an infected individual effectively contacts all its neighbors to expand the epidemics or information^37^.

For a given snapshot, a node *u* will be converted into infected one with a probability $P_u(t)$ at time *t*, we have,

4 $$\begin{aligned} P_u(t)=1-\prod _{v\in \Gamma _u}(1-\mu P_v(t-1)), \end{aligned}$$

where $\Gamma _u$ is the neighbors of node *u* and infection probability $\mu$ can be estimated by IAIP model (Iterative Algorithm for estimating the Infection Probability)^19^. For node *v* in Eq. (4), it is reasonable to assume $P_v(t)=1$ for infected node and $P_v(t)=0$ for susceptible or recovered node. Obviously, the initial condition is,

5 $$\begin{aligned} P_u(0)=\left\{ \begin{array}{l} 0\quad \text{ if } \text{ node } u \text{ is } \text{ susceptible } \text{ or } \text{ recovered }\\ 1 \quad \text{ if } \text{ node } u \text{ is } \text{ infected }\\ \end{array} \right. , \end{aligned}$$

By solving Eq. (4) under initial condition Eq. (5), $P_u(t)$ will be converged to a steady state denoted by $P_u(t_c)$ , where $t_c$ is the convergence time. The final score $P_u=P_u(t_c)$ is the probability to be infected of susceptible node while spreading achieves steady state. More precisely, we run the percolation according to Eq. (4) until the process dies, that is, for each node *u*, $P_u(t)=P_u(t-1)$ under given permissible error. Of course, we can also predict the probability of each node being infected at a certain step *t* after the snapshot.

In order to evaluate the performance of the proposed model, we use Pearson correlation $\rho$ between the result of averaging on *N* simulations and that of probability prediction model, that is:

6 $$\begin{aligned} \rho =\frac{N\sum _{i=1}^{N} x_i y_i-\sum _{i=1}^{N} x_i \sum _{i=1}^{N} y_i}{\sqrt{N\sum _{i=1}^{N} x_i^2-\left( \sum _{i=1}^{N} x_i\right) ^2} \sqrt{N\sum _{i=1}^{N} y_i^2-\left( \sum _{i=1}^{N} y_i\right) ^2}}, \end{aligned}$$

where $\overrightarrow{p}_r=(x_1,x_2,\ldots ,x_N)$ and $\overrightarrow{p}_e=(y_1,y_2,\ldots ,y_N)$ are the vector of infected probability of nodes obtained by simulations and by probability prediction model respectively, and *N* is the number of nodes of networks.
